# Adult-Generated Hippocampal Neurons Allow the Flexible Use of Spatially Precise Learning Strategies

**DOI:** 10.1371/journal.pone.0005464

**Published:** 2009-05-07

**Authors:** Alexander Garthe, Joachim Behr, Gerd Kempermann

**Affiliations:** 1 CRTD – Center for Regenerative Therapies Dresden, Dresden, Germany; 2 Department of Psychiatry, Charité University Medicine, Berlin, Germany; University of Alabama, United States of America

## Abstract

Despite enormous progress in the past few years the specific contribution of newly born granule cells to the function of the adult hippocampus is still not clear. We hypothesized that in order to solve this question particular attention has to be paid to the specific design, the analysis, and the interpretation of the learning test to be used. We thus designed a behavioral experiment along hypotheses derived from a computational model predicting that new neurons might be particularly relevant for learning conditions, in which novel aspects arise in familiar situations, thus putting high demands on the qualitative aspects of (re-)learning.

In the reference memory version of the water maze task suppression of adult neurogenesis with temozolomide (TMZ) caused a highly specific learning deficit. Mice were tested in the hidden platform version of the Morris water maze (6 trials per day for 5 days with a reversal of the platform location on day 4). Testing was done at 4 weeks after the end of four cycles of treatment to minimize the number of potentially recruitable new neurons at the time of testing. The reduction of neurogenesis did not alter longterm potentiation in CA3 and the dentate gyrus but abolished the part of dentate gyrus LTP that is attributed to the new neurons. TMZ did not have any overt side effects at the time of testing, and both treated mice and controls learned to find the hidden platform. Qualitative analysis of search strategies, however, revealed that treated mice did not advance to spatially precise search strategies, in particular when learning a changed goal position (reversal). New neurons in the dentate gyrus thus seem to be necessary for adding flexibility to some hippocampus-dependent qualitative parameters of learning.

Our finding that a lack of adult-generated granule cells specifically results in the animal's inability to precisely locate a hidden goal is also in accordance with a specialized role of the dentate gyrus in generating a metric rather than just a configurational map of the environment. The discovery of highly specific behavioral deficits as consequence of a suppression of adult hippocampal neurogenesis thus allows to link cellular hippocampal plasticity to well-defined hypotheses from theoretical models.

## Introduction

The last few years have seen progress in elucidating the relevance of adult neurogenesis for hippocampal function with respect to both learning and affective functions [1]–[8]. However, behavioral assessments in the Morris water maze (MWM), which despite some well-known disadvantages is considered a “gold standard” in the field and a prime test of more complex hippocampal function in spatial learning, yielded somewhat equivocal results [2], [4], [6], [9]. It has sometimes been suggested that the discrepancies were largely due to the different paradigms used to suppress adult neurogenesis (cytostatic drugs, irradiation, or genetic manipulation). Although we here also present a new, simple, and efficient way to suppress adult neurogenesis our main focus was rather on increasing the sensitivity of the water maze task to identify those qualitative changes in test performance that, according to our hypothesis, should be dependent on adult-generated neurons in the dentate gyrus (DG).

We hypothesize that adult neurogenesis optimizes the lean neuronal network of the DG to cope with changing, behaviorally relevant stimuli in the environment [10]. Survival in a changing environment requires balancing between establishing stable cognitive contingencies on one side and maintaining the possibility for flexibly altering these contingencies on the other (stability-plasticity dilemma). Our specific hypothesis, derived from a computational model, is that new neurons in the DG allow decreasing the risk of catastrophic interference between already encoded contingencies and newly appearing ones when the task conditions change [11], [12]. To test our hypothesis we used a reversal protocol of the classical water maze task, where the hidden platform is moved after three days of a first acquisition phase, whereas all cue configurations outside the arena remain unchanged [13]. To further support the formation of stable representations the starting positions remained constant for each day of the experiment. After changing the goal position animals are thus forced to re-learn their response to master the task successfully. In such situation a robust functional plasticity of the encoding network in the DG is required because an encoding rule acquired previously during the first acquisition phase has to be omitted in favor of a new one representing the changed cue-goal configurations.

To assess the qualitative aspects of learning, we analyzed the different behavioral strategies used to find the hidden platform in the Morris water maze [14]–[17]. Classification of searching behaviors and their associated swim patterns was performed using a parameter-based algorithm, which allows comparisons between the contributions of each respective strategy consistently against the same standard. Whereas we found control animals to proceed reliably towards using allocentric strategies, where distal cues provide geometric reference to an animal's current location, treated mice largely failed to do so. After platform reversal, a lack of newborn hippocampal neurons resulted in a strong persevering preference for the old goal position. Thus, our findings clearly point towards a highly-specific role for adult generated granule cells in mastering a spatial task flexibly and efficiently, especially when a previously acquired rule needs to be abandoned in favor of a new but still similar one.

## Results

### Suppression of adult neurogenesis

**Figure 1:**
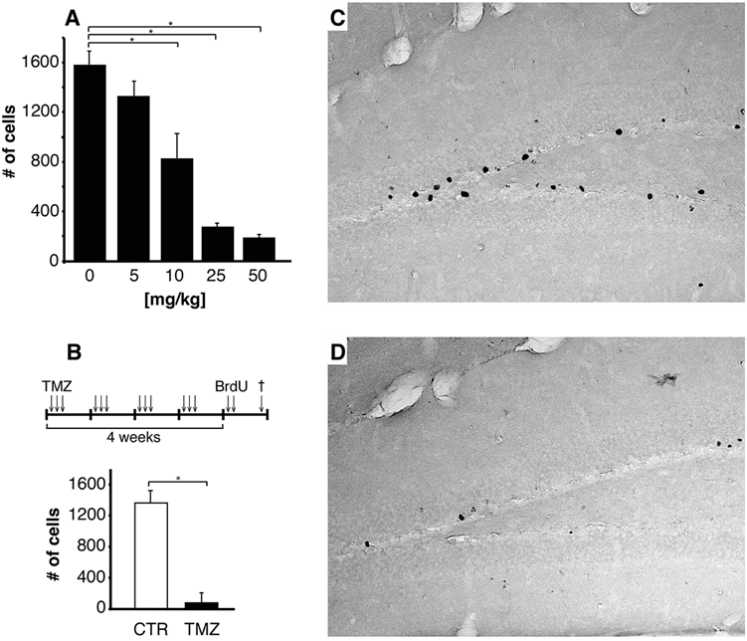
Effective suppression of adult hippocampal neurogenesis in C57BL/6 mice using temozolomide (TMZ). (A) Suppression of proliferation in the dentate gyrus was dosage dependent. After monocyclic treatment (one daily injection on three consecutive days) using 25 mg/kg TMZ total numbers of proliferating cells were reduced by more than 80%. Because doubling the dose did not result in significant lower numbers of BrdU^+^ cells, we used 25 mg/kg as the standard dose for all other experiments in this study. Proliferation was detected by incorporation of BrdU following a single injection using 50 mg/kg i.p. BrdU four days after end of TMZ treatment. (B) One treatment cycle consisted of single daily injections on three consecutive days followed by a resting period of four days. After four cycles of TMZ treatment proliferation was reduced by more than 90% (t(9) = 2.94, p<0.001). A single injection of BrdU was given four days after end of TMZ treatment. (C and D) BrdU^+^ cells in the dentate gyrus of mice after multicyclic treatment using either saline or TMZ (25 mg/kg), respectively.

To suppress neurogenesis in adult mice we established a new pharmacological approach based on four cycles of treatment with the DNA-alkylating agent temozolomide (TMZ; Figure 1). TMZ shows an excellent blood brain barrier passage and has a very advantageous side effect profile [18]–[20]. In a dosage finding experiment we found a clear dose-dependency after monocyclic treatment. Using 25 mg TMZ per kg body weight for three single daily injections was found to reduce proliferation (number of BrdU-positive cells) in the dentate gyrus by more than 80% (277±23 vs. 1589±134 cells in vehicle controls, t-test: t(9) = 3.14, p<0.05). Because doubling that dose did not result in a significant higher reduction of BrdU-positive cells TMZ was applied at a dose of 25 mg/kg for all following experiments. After four treatment cycles precursor cell proliferation was decreased by >90% (1347±178 BrdU^−^positive cells in controls vs. 84±93 for TMZ treated mice, resulting in a substantial reduction of recruitable newborn granule cells (t-test: t(9) = 2.94, p<0.001).

**Figure 2:**
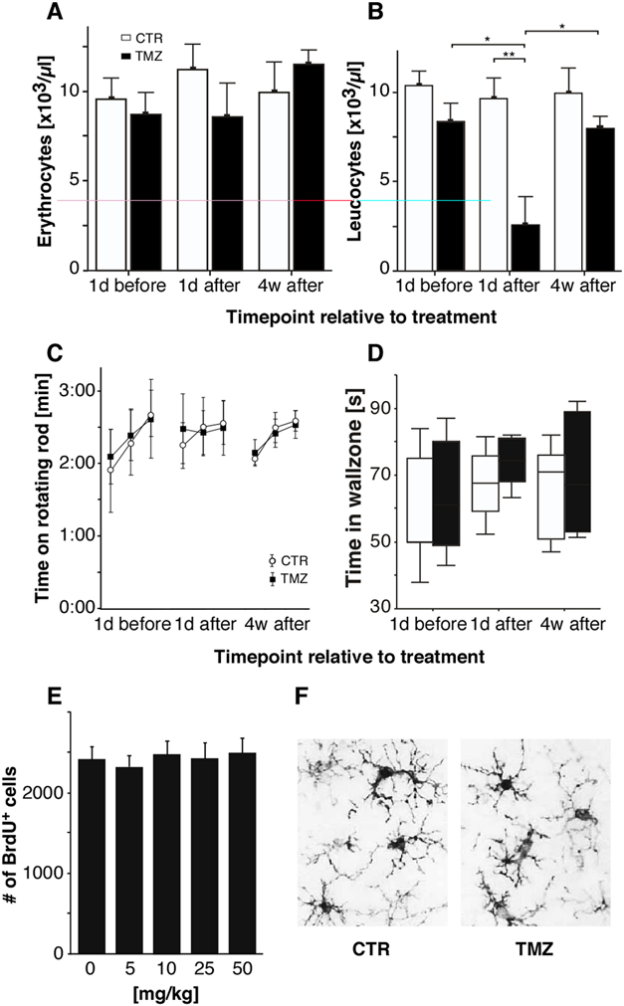
At the time point of behavioral testing no potentially confounding side effects were found. (A and B) As expected, hematology only revealed a significant leucopenia immediately after four cycles of TMZ (t-test: t(4) = 3.59, p<0.05). The number of leukocytes fully recovered after four weeks of resting. No differences were found for erythrocytes, hemoglobin and platelets. (C and D) Locomotor abilities and exploratory behavior appeared to be unimpaired after TMZ treatment. (E and F) Expression of Iba-1 was used to identify activated microglia in the dentate gyrus of C57BL/6 mice. No elevated numbers of Iba-1^+^ cells were found for any of the TMZ doses used in the dosage finding experiment indicating the absence of inflammatory processes. The right micrograph represents microglia in mice treated with 50 mg/kg TMZ.

In contrast, TMZ-induced myelosuppression had fully recovered after 4 weeks, when the animals were tested in the water maze (Figure 2A, B). Increase of body weight, locomotor skills and motor activity were all normal (data not shown and Figure 2C). No differences in exploratory behavior were found between groups (Figure 2D). There was neither morphological activation of microglia nor an increase in microglia numbers (Figure 2E, F). The 4-week interval thus ensured that at the time of behavioral testing only a minimum of potentially recruitable neurons would be present [21] but all potentially relevant transient side effects had ceased.

### Electrophysiology

**Figure 3:**
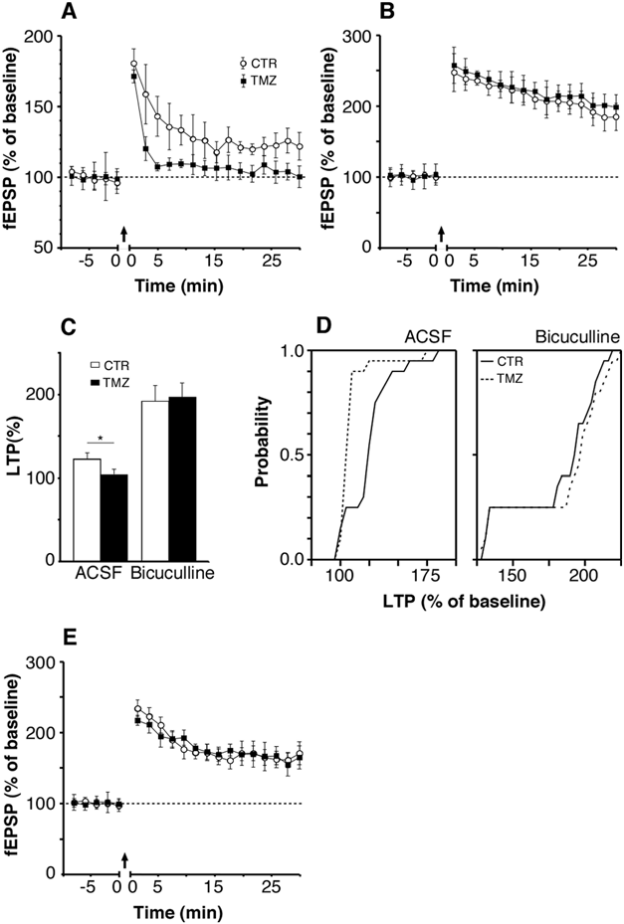
LTP in C57BL/6 mice with suppressed adult neurogenesis. (A) In TMZ mice treatment prevented LTP in the dentate gyrus of hippocampal slices perfused with ACSF, but LTP was induced in control mice (ANOVA F(1,198) = 252.8, p<0.05). Previous studies showed that new, adult generated granule cells facilitate the weak LTP observed, while LTP induction in mature neurons is blocked by GABAergic inhibition under physiologic conditions (16, 17). (B) Using GABA_A_ antagonist biculline a strong LTP was observed in both treated mice and controls. (C) Significant differences between TMZ mice and controls appeared only for ACSF-LTP. (D) A cumulative histogram for both ACSF (right) and bicuculline (left) perfused hippocampal slices. Dashed lines represent TMZ mice, solid lines controls. (E) LTP in hippocampal region CA1 was not affected by TMZ.

To assess hippocampal function at the systems level we investigated LTP in the DG and CA1. In the DG, LTP is physiologically inhibited by GABAergic interneurons. Still lacking that inhibition the immature adult-born neurons, in contrast to older granule cells, show a reduced threshold for LTP induction [22], [23]. Consequently, unless the GABAergic inhibition of granule cells is blocked, stimulating the medial perforant path with high-frequency bursts elicits a weak LTP in the DG that is based on the newly generated neurons [2], [24]. In TMZ-treated mice this characteristic weak LTP was not detectable (Figure 3A, C, D, F(1,198) = 252.8, p<0.05, ANOVA). After application of GABA receptor antagonist bicuculline, however, strong LTP could be elicited in both groups, indicating physiological synaptic plasticity in mature neurons (Figure 3B). LTP in CA1 was unaffected by TMZ treatment (Figure 3E). This implied that after TMZ treatment, only LTP in the DG was influenced and only in a manner consistent with the reduced level of ongoing neurogenesis.

### Reversal learning in the Morris water maze

**Figure 4:**
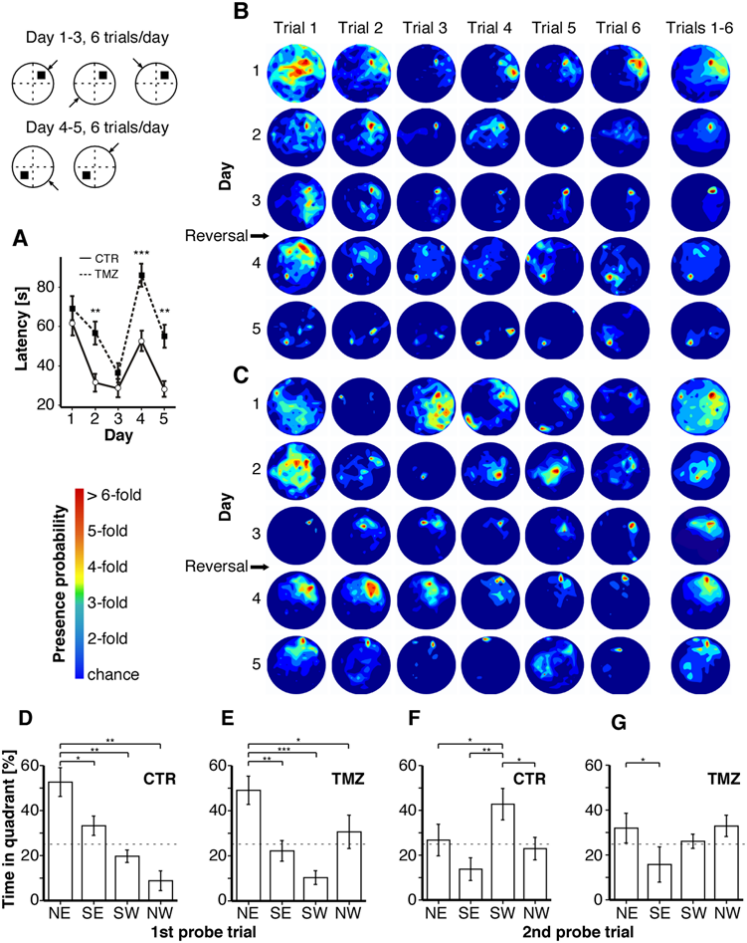
Spatial learning in C57BL/6 mice with suppressed adult neurogenesis. (A) Both groups successfully learned to navigate to the hidden goal, but TMZ treated mice needed longer to locate the hidden platform. While the TMZ group reached comparable latency times at the end of the first acquisition phase, mice with suppressed adult neurogenesis performed worse both transiently before and consistently after reversal. (B) For controls, the occupancy plot shows the rapid development of a place-specific preference for the platform position both before and after reversal. (C) In TMZ treated mice learning a correct place-specific response for the first goal position was significantly delayed but still effective. After platform reversal mice with suppressed adult neurogenesis showed a profoundly perseverating preference for the previous platform position. (D–G) Probe trials indicated successful spatial learning of the first goal for both groups. After reversal treated animals failed to develop a preference for the new goal quadrant.

We trained mice on a reference memory version of the MWM task with 6 trials per day for 5 days and a new platform position (reversal) on the beginning of day 4 [13]. Although TMZ-treated mice performed significantly worse than controls for the entire experiment (latency, F(1,590) = 34.95, p<0.001, repeated measures ANOVA), both TMZ-treated mice and controls (CTR) successfully learned to navigate to the hidden platform during the first three days (Figure 4A). At the end of this period there were no significant differences between the groups in path length and latency time to reach the hidden platform. Although TMZ-treated animals and controls generally succeeded in learning the MWM, significant differences appeared on day two of the acquisition phase regarding both latency and path length (Figure 4A, t(9) = 3.42 p<0.001 for latency and t(9) = 2.77, p<0.01 for path length, Student's *t*-test).

Swim speed of both groups did not differ throughout the experiment (data not shown). In the 1st trial of day 4 (1st probe trial), when the platform had been moved to the opposite quadrant, mice from both groups spent significantly more time in the previous goal quadrant than in the other quadrants, indicative of successful spatial learning (t(9), p<0.05 for comparison of the NE quadrant with each other quadrant and for each group, Student's t-test, Figure 4D, E).

After reversal, further significant differences were found. As apparent in the heat maps visualizing the probability for the mice to be present at a given location, TMZ-treated mice took significantly longer to develop a clear preference for the first goal and persevered swimming to that platform position even after two days of training following reversal indicating a lack of cognitive flexibility to cope with the altered challenge (Figure 4B, C). A second probe trial at the end of the reversal phase showed that, whereas controls had successfully learned to navigate reliably and precise to the new platform position, TMZ-treated mice largely failed to adapt to the changed situation and to develop an appropriate new spatial preference (Figure 4F, G).

### Qualitative aspects of spatial learning

**Figure 5:**
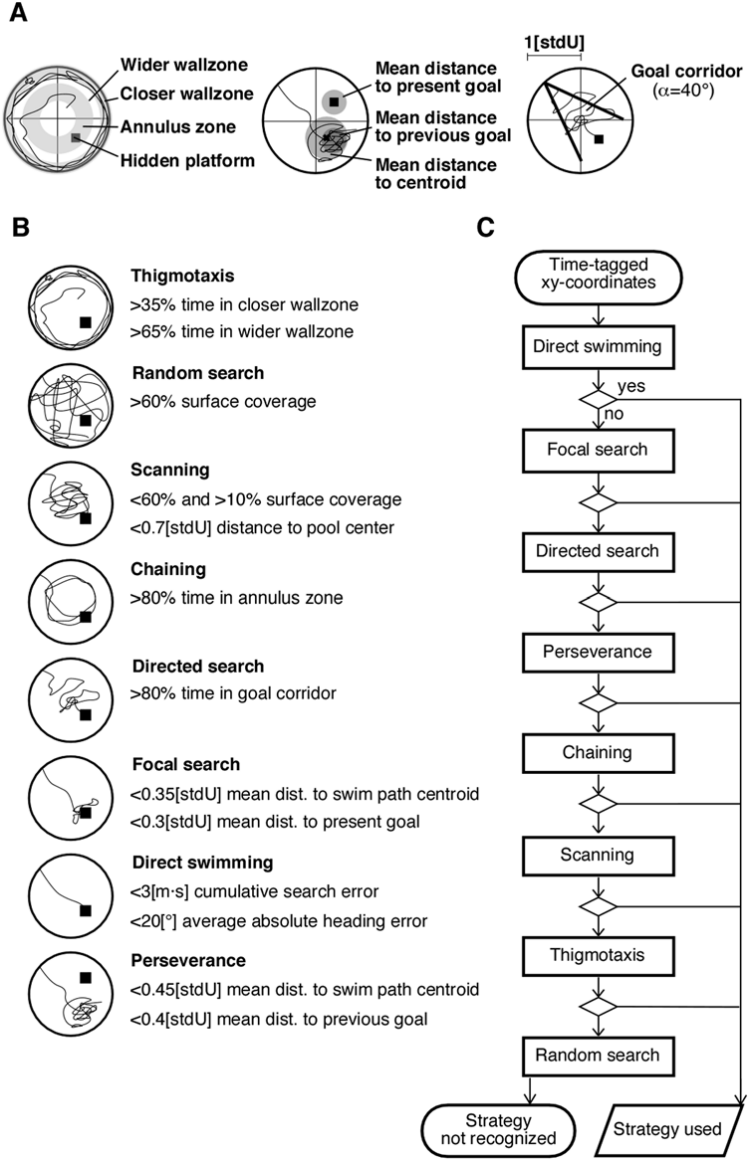
Algorithm-based classification of search strategies. (A) Determination of variables used in the classification process. The pool was divided into distinct zones to calculate the amount of time spent in the respective areas (left). Average distance of all datapoints of a given swim path to its centroid and to the present or previous goal position was used during the classification process (middle). Search patterns based on a directional preference for the goal position were identified using a triangular shaped corridor expanding from the starting point with its bisecting line towards the platform (right). (B) Each strategy was identified by one or two parameters representing their respective abstract key properties. (C) Because some search patterns were defined less specific than others exclusion of strategies had to be achieved in a particular order. Search patterns not recognized were classified by hand.

**Figure 6:**
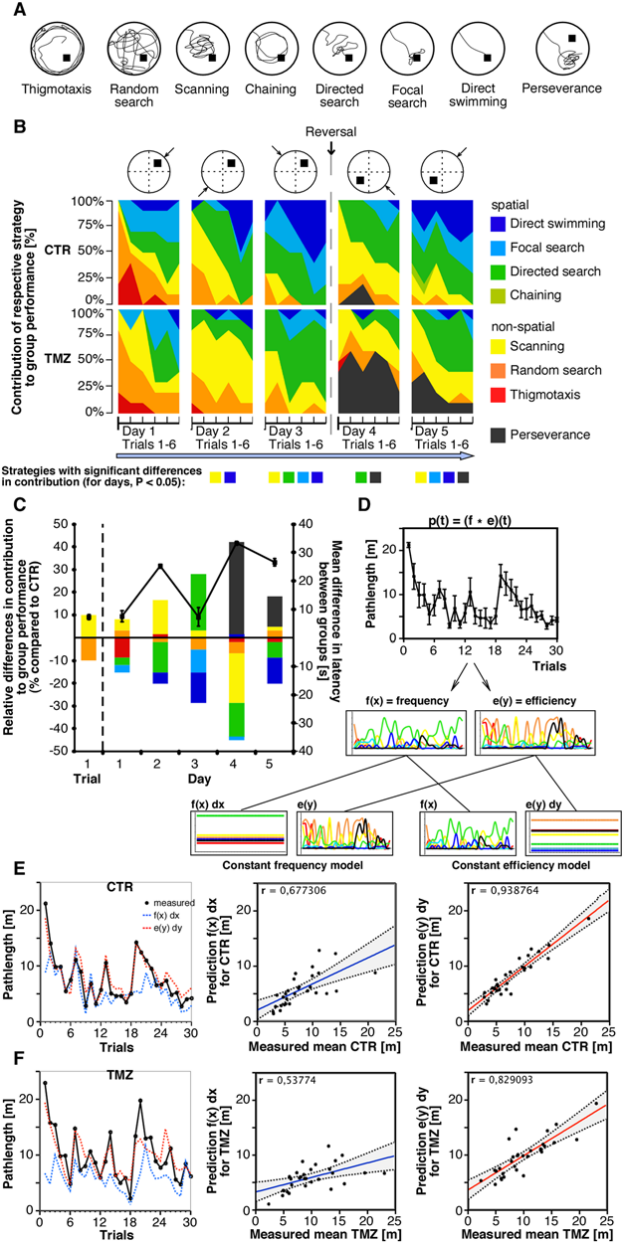
Qualitative analysis of spatial learning in mice with suppressed adult hippocampal neurogenesis. (A) Examples of search strategies recognized by the classification algorithm used. (B) Both groups showed a clear progression towards increasingly hippocampus-dependent strategies. The top row represents the basic experimental protocol including the hidden platform position and the four starting positions used (arrows). Contribution of respective strategies to group performance in learning the MWM task was analyzed by classifying all trials using a parameter-based algorithm (Figure 5). Strategies are color coded. See text for statistical comparisons. (C) Compared to controls in treated animals respective strategies contributed differentially to group performance. Differences are shown as percent of contribution relative to controls. The color code used is the same as in (B). The black line shows the mean difference between groups in latency to reach the hidden platform. (D) Principle of convolution analysis according to Brody et al. [14]. (E and F) Comparison of measured mean path lengths with predictions derived from convolution analysis using a constant-frequency model and a constant-quality model. An efficient progression towards directed or place-specific navigation strategies was found to be the general underlying learning scheme in both groups.

To assess the qualitative aspects of learning the water maze task we analyzed the respective search strategies displayed by the mice to locate the hidden platform (Figure 5). Parameter-based classification of search patterns turned out to be reliable for both groups indicating that treated animals used identical basic principles to implement the full range of strategies (Figure 6A).

Before and after reversal we found a clear progression from thigmotaxis to direct swimming for both groups (Figure 6B; thigmotaxis F(4,72) = 6.37, p<0.001, random search F(4,72) = 11.17, p<0.001, scanning F(4,72) = 6.14, p<0.001, directed search F(4,68) = 3.95, p<0.001, focal search F(4,72) = 8.63, p<0.001, direct swimming F(4,72) = 7.8, p<0.001 ANOVA, inner-subject test). However, treated animals and controls differed significantly in using the respective strategies to master the task. The inter-subject test revealed highly significant differences for focal search (F(1,18) = 14.82, p<0.001) and direct swimming (F(1,18) = 23.31, p<0.001) as well as significant differences for scanning (F(1,18) = 5.78, p<0.05) and perseverance (F(1,18) = 9.87, p<0.05). A highly significant interaction effect day×group was found for direct swimming (F(4,72) = 6.67, p<0.001) and perseverance (F(4,72) = 8.34, p<0.001). For scanning (F(4,72) = 3.17, p<0.05) and directed search (F(4,72) = 2.66, p<0.05) the interaction was significant. Group specific differences firstly appeared only on the level of scanning, when the search becomes increasingly systematic. Importantly, the group differences turned out to be especially large when hippocampus-mediated spatial precision became essential. Over time, trial-wise contribution of strategies differed significantly between treated mice and controls for scanning (p<0.05, Mann-Whitney on trials 3, 11, 12 and 13), focal search (p<0.05 on trial 14), directed search (p<0.05 on trials 10, 17 and 18), direct swimming (p<0.001 on trials 11, 16 and 17) and perseverance (p<0.05 on trials 19, 20, 22 and 23). All ANOVAs were done using the Bonferroni correction.

Because the distribution of differences in strategy use appeared to reflect an underlying temporal pattern we looked for systematical changes in the respective contributions to group performance in treated animals compared to controls (Figure 6C). In the first trial treated mice and controls differ only to a small degree with respect to random and scanning strategies. Importantly, when exposing the animals to the pool for the very first time no differences were found for thigmotaxis behavior (Figure 6C left). In general, the use of directed and place specific search patterns turned out to be at least delayed in mice with suppressed adult neurogenesis. Furthermore, to find the hidden platform TMZ mice consistently relied on scanning and directed search at the expense of focal search and direct swimming, the latter representing the truly allocentric search patterns with the highest spatial precision. On day 3, in the late phase of the first acquisition period, both focal search and direct swimming were found only sparsely among treated animals. Surprisingly, on that day the absolute mean difference in latency between groups was found to be minimal, whereas the sum of relative differences in strategy contribution was high for TMZ mice.

### Convolution analysis

Our finding that treated mice reached comparable performance levels at the end of the first acquisition phase raised the important question whether progression to the spatially most precise, allocentric strategies is mandatory for learning the reference memory version of the water maze. Alternatively, the task might be mastered by simply improving the proficiency of using generally less effective strategies. Therefore, we used convolution analysis to test the hypothesis that effectively switching to the most effective search patterns is the learning strategy generally underlying acquisition of the MWM task (Figure 6D–F, see methods section for details).

By comparing the values measured with predictions from two models holding constant either the frequency or efficiency with which each strategy was applied, linear regression analysis revealed an effective progression towards direct swimming to be the general learning strategy in both groups (CTR: r = 0.94 compared to r = 0.68; TMZ: r = 0.83 compared to r = 0.54; Figure 6 E, F). This finding indicates that treated mice, too, solved the task by trying to switch to the more advanced spatially precise learning strategies.

## Discussion

The key finding in our study is that suppression of adult hippocampal neurogenesis results in specific and subtle learning deficits in the reference memory version of the MWM task. Because of the notably high specificity of the deficits and our data on a normal hematological, physiological and behavioral status at the time of testing, the observed effects of a substantially reduced adult neurogenesis cannot be explained only as side effects of TMZ treatment. Importantly, because relying on the strict evaluation of numerically defined parameters the algorithm-based classification of search patterns served as a sensitive internal control for general changes in learning behavior. The reliability with which respective search patterns were identified for treated mice did not differ from that found for the control group, indicating that TMZ treatment did not disrupt the constitutive elements necessary to develop the full range of search strategies.

Six to 8 week-old adult-generated granule cells become selectively recruited into existing hippocampal networks while learning the spatial version of the MWM task [21]. Therefore, in order to effectively remove the cells usually recruited in the course of spatial learning, both duration of TMZ treatment and the time-point of behavioral testing after the end of treatment are of some importance to the study design. Using a multi-cyclic treatment paradigm over four weeks followed by a reconstitution period of another four weeks we ensured that only a minimum of new neurons was available for recruitment when mice were subjected to behavioral testing. The remaining adult-born granule cells would make only a minor functional contribution to information processing in the DG and thus their benefit might have been insufficient to allow effective coping with changes among stimulus configurations. Given that full reconstitution of the precursor cell pool likely occurs not within days, the role of new neurons born after the end of TMZ treatment could also be neglected.

Although TMZ did not completely wipe out adult neurogenesis (as reported after irradiation), treated mice showed profound impairments in cognitive flexibility while learning the MWM, both before and after platform reversal. During the first acquisition phase treated animals needed longer to develop a precise spatial preference for the goal. Furthermore, they relied primarily on a delayed progression to directed search patterns and generally failed to proceed to the most precise place-specific and allocentric strategies. Such selective impairment during task acquisition is consistent with data from a previous ablation studies [6], [7] and our own strain comparison study regarding the latencies to find a hidden platform [25].

After moving the goal to the opposite pool quadrant treated mice displayed profound difficulties to learn the platform's new position. The persevering preference for the first goal was abandoned only very slowly over a number of trials and even persisted on the second day after reversal. Nonetheless, convolution analysis clearly showed that the general pattern of progression towards more effective and directed strategies still exists in treated mice. Although the second probe trial indicated no successful learning of the reversed platform position, at least 50% of the TMZ mice still developed directed search patterns in favor of the new goal position. Therefore, as already found for the first acquisition phase, learning was not generally impaired in treated animals but appeared to be significantly delayed, when a change of the task's key aspects required the already learned contingencies to be changed.

Importantly, the inability to flexibly cope with changes among relevant contingencies as a consequence of substantially reduced numbers of adult generated hippocampal neurons is predicted by a recent mathematical model [12]. Following that model, to ensure the efficient and non-interfering storage of both newer and already existing representations in CA3, the encoding mechanism in the DG must be adapted depending on the respective statistical properties of the given spatial context. Consequently, in our study the lack of newborn and plastic granule cells prevented an effective adaptation of the DG network, especially after platform reversal when a main contingency has changed.

An effective progression from thigmotaxis to precise and place specific allocentric navigation depends on a metric representation of the environment which in turn requires a successful integration of the available distant cues. Thus, as the delayed progress to the most effective search patterns was consistently accompanied by a profound lack of spatial precision in getting to the goal most directly, it seems reasonable to assume that both impairments might have emerged from a single common effect of an impaired adult hippocampal neurogenesis. Indeed, deficits related either to spatial precision or reduced functional plasticity after reversal can be linked by the hypothesis that new adult generated granule cells serve to flexibly encode novel stimulus configurations experienced the first time. In order to provide a precise metric representation of the visual cues in terms of exact angles and distances allowing the animal to use true allocentric strategies, the relative positions between all relevant stimuli need to be integrated into a single coherent map during training. Since it has been shown by Goodrich-Hunsaker et al. that a specific function of the DG is to provide a metric representation as opposed to a just configurational one in CA3, the role of new granule cells might be thought of as a possible substrate for facilitating the rapid encoding and integration of novel cue configurations encountered by an organism when the relevant context has changed [26]. This interpretation is in accordance with findings from a recent study by Dupret et al. showing that ablation of adult neurogenesis results in an impairment to flexibly express spatial memories acquired previously using either changing or constant starting positions [7]. The essential need of an opportunity to form stable, integrated representations in order to develop allocentric search strategies is also consistent with our finding that in the control group focal search and direct swimming were used by more than 50% of the animals only after more than two days of training. Importantly, a repeated exposure to the same common spatial context (i.e. the MWM pool, including the hidden goal) seems to be an essential prerequisite for a detailed metric representation to be formed. The use of multiple starting positions, held constant within days likely supports the further formation of stable encoding rules for redundant stimulus configurations. This might at least partly explain the somewhat contradictory results of previous studies using the DMP version of the MWM paradigm without assessing the qualitative aspects of spatial learning [27], [28] (but see also [7]). In that variant, the position of the platform is changed in each trial, thus effectively preventing a stable platform-related encoding rule to become manifested in the DG network, which – as demonstrated here – requires more than one day or six trials of training. Only stable or at least reliably reoccurring variants of certain cue-configurations might allow the network in the DG to adapt sufficiently to perceive a change of the cues as significantly new.

Given an environment with changing contingencies, the lack of adult-generated granule cells in the DG would not only impair the animal's ability to encode novel aspects of the cue configurations encountered. Rather, both a clear differentiation between stimulus configurations being part of distinct sets as well as the integration of those belonging to the same sets of cues might be seriously compromised. Thus, the inability to develop true allocentric search strategies and a profound lack in flexibility after changing the platform's position may likely turn out to be two sides of the same medal but this link needs to be clarified in further studies.

The involvement of altered executive functions as a consequence of TMZ treatment needs to be discussed as an possible alternative explanation for the impairments found in our study. However, our results clearly argue against such an explanation. First, treated mice successfully suppressed their tendency to swim fast and repetitively along the wall, panicking to find a way out. From an executive perspective this can be considered as a key point for mastering the water maze task effectively. Indeed a wide range of pharmacological treatments, selective lesioning of brain regions and gene knock-outs resulted in substantially elevated levels of thigmotactic behavior which is often accompanied by passive floating [16], [29]. Because we did neither observe passive floating at all nor did we find any differences in thigmotaxis, a general effect of TMZ treatment on executive functions seems unlikely. Secondly, we have conducted a convolution analysis to show that mice from the TMZ group primarily relied on an effective progression through the strategies available. The fact that after a multi cyclic TMZ treatment the animals showed a clear dependence on the ability to switch their behavioral strategies towards the more effective hippocampal ones (as observed for controls) provides another strong argument against confounding side effects of TMZ on the executive system. However, as the general progression appeared to be slowed down after treatment potential side effects on executive functions cannot be completely ruled out. On the other hand it seems hard to explain why a problem in executive control caused as a side-effect of a systemic treatment should result in the selective inability to develop and use precise allocentric behavioral strategies.

Given the hypothesis that adult generated granule cells help the DG to avoid suffering from catastrophic interference our results appear to be in clear accordance with a central prediction of our computational model [12]. In that computational simulation a model organism was subsequently exposed to different environments for a sufficient time to show adaptation. The reversal paradigm used in our study implements the same concept for living animals and into the framework of a well-characterized hippocampus-dependent learning task. As predicted, treated animals largely failed to adapt efficiently when the task's contingencies changed following platform reversal. On the other hand the more general inability to develop fully allocentric search strategies was not predicted but this can be probably ascribed to the model's simplicity. Although the model predicts the occurrence of catastrophic interference in the absence of adult neurogenesis, the specific phenotype of living animals remains dependent on many, largely unknown features of information processing in the DG.

Our findings also need to be further discussed in the context of alternative ideas for specific functions of adult neurogenesis [30]–[35]. It has been suggested that a neuronal turn-over situation might help the hippocampus to forget old memories in favor for newer ones [36]. At first glance our results support such a view because difficulties to cope efficiently with the platform's reversal could be interpreted as simply being unable to forget the goal's previous position. However, to master the task it is not required to forget the old platform position. Failure to apply precise allocentric behavioral strategies is not explicitly predicted by such a model because development of allocentric representations appears to involve the integration rather than exclusion of information experienced over time.

A very detailed model of adult neurogenesis emphasizes the potential role of new neurons in the encoding of temporal information (“time-stamp model”) [30], [31]. At present the temporal resolution of our experiment is too low and our knowledge about the actual stimuli between which the temporal association would have to be formed is to limited for addressing hypotheses from that model directly. In any case, adult-generated granule cells might serve multiple and distinct functions at different stages of their differentiation and maturation. Therefore, the different models – including our own model, from which we derived our hypothesis [12] – might each cover particular functional aspect of adult neurogenesis. A “grand unifying theory” will have to integrate these different aspects into one complete model.

The effectiveness of TMZ in suppressing adult neurogenesis and the implication of this suppression on hippocampal performance were welcome in the context of our study. For the clinical context, however, our data might raise some concern about lasting cognitive side effects of anti-proliferative treatment that will certainly deserve further investigation.

## Materials and Methods

### Animals and tissue preparation

For all experiments we used 6–8 weeks old female C57BL6 mice. To label adult generated cells animals received one single intraperitoneal injection of bromodeoxyuridine (BrdU, 50 µg/kg body weight, Sigma). Tissue preparation was done as described [37]. The mice were kept at the animal facility of the Max Delbrück Center for Molecular Medicine (MDC) Berlin-Buch, Germany. All local and federal regulations of animal welfare in research were followed. The experiment was approved by the responsible authority, Landesamt für Technische Sicherheit und Gesundheit (LaGetSi) Berlin.

### Drug treatment

Temozolomide (TMZ, Temodal®, SP Europe, Belgium) is an alkylating agent intended for the treatment of recurrent malignant glioma. In a dosage finding experiment we found a dose of 25 mg/kg body weight to be effective in suppressing adult neurogenesis by more than 80% after monocyclic (3 days) of treatment (Figure 1A).

Consequently, to suppress adult neurogenesis, mice from the treatment group (TMZ) received injections of TMZ at 25 mg/kg (i.p., 2,5 mg/ml in 0.9% NaCl), whereas the control group (CTR) received sham injections of saline only. This regimen was given on the first three days of a week for 4 weeks to resemble paradigms used for glioma treatment in humans (Figure 1B). Behavioral testing was performed 4 weeks after the final TMZ injection.

For adult-born granule cells to become recruitable the sequence of proliferation, differentiation and maturation requires approximately up to 28 days. Thus, suppressing adult neurogenesis for at least 4 weeks combined with a reconstitution period of 4 more weeks ensured that most of the cells borne immediately before onset of TMZ treatment would have been already used or eliminated by apoptosis. Using intercalating convalescence times, we minimized the risk of confounding side effects during behavioral testing.

It has recently been shown that 6–8 weeks old, adult generated granule cells become selectively recruited during acquisition of a spatial learning task [21]. Therefore, our primary aim was to minimize the number of potentially recruitable adult-born neurons exactly at that time, when the new granule cells should be of particularly high relevance for task acquisition. Because we suppressed proliferation for at least 4 weeks and behavioral testing began an additional 4 weeks later, at the time-point of the behavioral analysis the subpopulation of 5–7 week old adult generated neurons is primarily affected by TMZ. An immediate recovery of the stem cell niche seemed unlikely as it was found that after wiping out proliferation in the SGZ by irradiation reconstitution occurs only slow and on a prolonged timescale [38]. Consequently, at the time, when the mice were learning the water maze task only very few (or no) new granule cells were available to be functionally integrated into existing circuits.

### Immunohistochemistry

Histology procedures were performed as described previously (*2*). Briefly, animals were perfused with 4% paraformaldehyde, the brains removed from the skulls, cryoprotected and sectioned at 40 µm on a dry ice-cooled sliding microtome. For immunostaining we used rat anti-BrdU (1∶500, Biozol), goat anti-Iba1 (1∶250, SantaCruz) as primary and biotinylated-anti-rat (1∶500, Dianova) as secondary antibodies.

For visualization of BrdU incorporation, DNA was denatured in 2N HCL for 30 minutes at 37°C. Free floating sections were then rinsed in 0.1 M borate buffer, pH 8.5, and thoroughly washed in tris-buffered saline (TBS), pH 7.4. To block endogenous peroxidase reactions, sections were pretreated with 0.6% H_2_O_2_. Sections were incubated with primary antibody against BrdU in TBS supplemented with 0.1% TritonX-100 and 3% donkey serum (TBS-plus) at 4°C overnight. After rinsing the sections with TBS and a blocking step with TBS-plus, incubation with the biotinylated secondary antibody (1∶500, Dianova) in TBS-plus followed. ABC reagent (Vectastain Elite, Vector Laboratories) was applied for 1 hour at a concentration of 9 µl/ml per reagent. Diaminobenzidine (DAB, Sigma) was used as a chromogen at 0.25 mg/ml in TBS with 0.01% H_2_O_2_ and 0.04% nickelchloride followed by rinsing with tap water and TBS. The sections were mounted on gelatine-coated glass slides and coverslipped with Neomount.

The number of BrdU-positive cells was analyzed following the standard routine of our laboratory. The method is a simplified version of the optical fractionator principle. In a complete series of 40 µm sections, 240 µm apart, BrdU-positive cells in the SGZ and the granule cell layer were counted exhaustively, but cells in the uppermost focal plane (at 40× magnification) were disregarded to avoid oversampling at the cutting surfaces. All counts were done with the experimenter ignorant of the treatment group of the specimen. A total of 10 animals were used.

### Hematology

Blood cell counts were obtained using a Beckman Coulter Ac-T Diff Hematology Analyzer with a software version for veterinary applications. After anesthetizing the animals with diethylether, blood samples were taken from the retro-orbital sinus. Blood samples were taken 1 day before, 1 day after and 4 weeks after treatment and collected using EDTA coated MiniCollect® tubes (Greiner bio-one, Kremsmünster, Austria). A total of five animals were used.

### Electrophysiology

350 µm-thick horizontal slices containing the entorhinal cortex, the subiculum, and the hippocampus were prepared from 3 to 4 months-old female mice. The slices were transferred to an interface recording chamber continuously perfused with an aerated (95% O_2_, 5% CO_2_), prewarmed (32°C) artificial cerebrospinal fluid (ACSF) containing (in mM) NaCl 129, Na2PO4 1.25, NaHCO3 26, KCl 3, CaCl2 1.6, MgSO4 1.8, glucose 10 at a pH of 7.4. After 2 h of equilibration the medial perforant path was stimulated and the evoked potentials in the dentate gyrus were recorded in the molecular layer above the upper blade by using a glass capillary microelectrode filled with artificial cerebrospinal fluid (tip resistance 2–3 MΩ). Microelectrodes were prepared from borosilicate glass tubes. After 10 min of stable baseline response to test stimulation (once every 30 s), the ability to elicit LTP was assessed. To induce LTP, four tetani of high-frequency stimulation were applied at 100 Hz for 1 s with 10 s intertrain intervals. Responses were recorded every 30 s for 30 min after LTP induction. In a parallel set of experiments, the ACSF contained 5 µM bicuculline (BCM, Sigma, Deisenhofen, Germany) to block GABA_A_ receptor-mediated inhibitory activity.

To record field EPSPs (fEPSPs) in the CA1 region of the hippocampus, afferent fibers of the Schaffer collateral pathway were stimulated and recordings were made in the CA1 pyramidal cell layer. For LTP experiments, a 10-min baseline was recorded by stimulating every 30 s. LTP was induced by using a 100 Hz stimulation (four trains, 1 s in duration), after which responses were elicited once every 30 s at the same stimulation intensity for 30 min. Signals were filtered at 3 kHz and sampled at 10 kHz using a TIDA interface card (HEKA, Lambrecht, Pfalz, Germany).

All data were analyzed offline using TIDA software. Amplitudes of evoked field potentials were measured from an average of 4 peaks. Data were expressed as means±SEM and statistical comparison was done by repeated-measures ANOVA on the last 10 min (Aabel Software, Gigawiz). Significance level was set to *p*<0.05.

### Rotarod and open field task

The rotating rod apparatus (Columbus Instruments) was used to measure the ability of the animals to improve their locomotor skills with training [39]. 1 day before, 1 day after and 4 weeks after TMZ/sham treatment exploratory activity was evaluated using an open field test [40].

For the rotarod task mice were placed on the rod (3 cm in diameter) for three trials per day for three consecutive days. Each trial lasted a maximum of 5 min, while the rotating rod underwent a linear acceleration from 4 to 40 rpm. Animals were scored for their latency to fall for each trial. Between the trials the animals rested a minimum of 10 min to avoid motoric fatigue. All animals were subjected to the rotarod task 1 day before, 1 day after and 4 weeks after TMZ/vehicle treatment.

For the open field test each animal was placed in the center of a white plastic chamber (60×60×20 cm) under standard room-lighting conditions. Overall activity and the time spent by each animal in the center and/or the wall zone was measured and analyzed using a digital camera on the ceiling operated via Ethovision (Noldus, Netherlands). The time spent in the center was used as an index of general anxiety levels.

### Morris water maze task (MWM)

Two weeks after end of treatment, mice were trained in the reference memory version of the Morris water maze task [41] to locate a hidden escape platform in a circular pool (1.89 m diameter). Water was made opaque with non-toxic white paint and kept at a temperature of 19–20°C. Each mouse was given 6 trials a day for 5 consecutive days with an inter-trial interval (ITI) of 30 mins. Platform position was changed after day 3 (Figure 4). Mice were released from one of four possible starting points and allowed to search up to 120 s for the platform. During each day the starting position remained constant. Irrespective of trial performance mice were guided to the platform and allowed to remain there for at least 15 s. Swim paths were recorded using Ethovision (Noldus) and further analyzed using Matlab (The Mathworks, USA).

### MWM data analysis

General spatial learning was analyzed using classical parameters like latency to reach the platform, swim path length and relative time spent in the four quadrants (Figure 4).

To characterize the development of a spatial preference for the goal platform the MWM pool was divided into 10×10 cm wide sectors allowing the presence probability of an animal in each sector to be represented as heat map-like occupancy plots.

To assess the qualitative aspects of learning the MWM task we analyzed the search strategies used by the animals to locate the hidden platform. Originally, the existence of different behavioral strategies in the context of spatial learning was demonstrated by Wolfer and Lipp [14], [16], [17], [42]. Here the concept of different search strategies was modified as shown in Figure 5. Briefly, swim path data from Ethovision (Noldus, NL) were used to derive the time-tagged xy-coordinates for classifying the respective predominant search strategies by an algorithm implemented in Matlab. Classification criteria served an only descriptive function and were not meant to prove the involvement of particular cognitive processes or brain structures.

In the course of learning the MWM, the animals showed a sequential use of different search patterns ranging from initially almost undirected to spatially precise, highly efficient and allocentric strategies. Immediately after being introduced to the MWM pool the first time some animals showed a behavior known as “wall hugging” or “thigmotaxis”. Thigmotaxis is considered to be a first and highly emotional response to a new and stressful situation. Usually thigmotaxis is rapidly overcome and replaced by the “random search” strategy covering the entire pool surface. As the animals gathered more knowledge of the MWM arena their search behavior became increasingly restricted to the central pool area where the availability of distant visual cues is maximal. Because in this stage the mice scan the environment for landmarks this strategy is called “scanning”. For both random search and scanning any spatial or even directional preference is absent. “Chaining” is proposed to be a successor of scanning where the animals show a clear preference for the goal annulus having learned the correct distance of the goal platform to the wall. Once the animals develop a directional preference the use of distant visual landmarks is evident and thus their behavior albeit still being egocentric has become hippocampus-dependent. This point is marked by the “directed search” strategy where the search becomes directional restricted in a triangular fashion pointing from the starting position towards the actual goal. Although path lengths swum may be initially not significantly shorter than for the scanning or chaining strategy directed search is usually the beginning of a subsequent refinement in the efficiency to reach the goal platform from each possible start position. This phase has to be considered as transition from egocentric to allocentric navigation. Therefore the two most efficient behavioral strategies to navigate to the hidden platform are “focal search” where the animals spend most of the time in the nearest neighborhood of the goal and “direct swimming” where they navigate directly to the goal regardless of the actual starting position used. The development of a precise and place specific preference depends on the integration of the relevant cues into an allocentric cognitive representation. Principally “perseverance” can be found among all groups irrespective of treatment and represents a still present preference for the previous goal position until the animal's behavior is changed as a sign of functional plasticity.

The classification process was implemented as a script in Matlab and relied on a set of numerical parameters (Figure 5) and applied those criteria to each trial of the MWM experiment therefore providing an unbiased objective method to identify certain qualitative properties of the recorded swim paths. All criteria represent the common abstract properties of the respective strategy and correspond directly to their special functional demands. To avoid misclassifications due to shared features among multiple strategy definitions the classification process excludes the respective patterns serially. Thus search patterns all of which are enclosed by other patterns as special cases have to be sorted first followed by the less precisely defined ones. The algorithm used in our study was tested on 4320 example trials showing an overall reliability of more than 90% (trials not recognized at all were interpreted as errors).

### Convolution analysis

Generally, mice can use different higher-level strategies to learn the MWM task. As an alternative to an effective progression towards the principally most precise search strategies animals could also just improve their proficiency in applying less precise strategies. Which of those higher-level strategies is preferentially used makes a fundamental difference for interpreting the true hippocampal learning. Especially under the highly reductionistic conditions found for the MWM task just practicing non-spatial strategies such as scanning or chaining can result in comparable latency times without any contribution of hippocampus-specific behaviors. Therefore we evaluated the respective contribution of both general strategies using convolution analysis [14].

Learning performance in a spatial task is usually measured using parameters like latency or path length. Common to those parameters is their dependence on more basic variables that cannot be easily assessed directly. Accordingly both latency and path length can be interpreted as values of convoluted functions. Given the concept of distinct qualitative search strategies, learning performance in the MWM depends on the respective frequency with which a respective strategy is applied (function *f(x)*) and the quality that is found in applying a certain search pattern as measured by the respective path length for that pattern (function *e(y)*). Thus for the animals' task performance we get p(*t*) = (*f* * *e*)(*t*) as the convoluted function of *f(x)* and *e(y)*.

To estimate the relative contribution of each respective function to the learning performance p(*t*) we compared the measured path length for each search strategy (p(*t*)) with a prediction derived from a model where either the frequency (*f(x) dx*) or quality (*e(y) dy*) is held constant for the whole experiment. The respective means of frequency and path length over all days were used as constants to calculate those predictions (Figure 6D).

## References

[1] Santarelli L, Saxe M, Gross C, Surget A, Battaglia F (2003). Requirement of hippocampal neurogenesis for the behavioral effects of antidepressants.. Science.

[2] Saxe MD, Battaglia F, Wang JW, Malleret G, David DJ (2006). Ablation of hippocampal neurogenesis impairs contextual fear conditioning and synaptic plasticity in the dentate gyrus.. Proc Natl Acad Sci U S A.

[3] Shors TJ, Miesegaes G, Beylin A, Zhao M, Rydel T (2001). Neurogenesis in the adult is involved in the formation of trace memories.. Nature.

[4] Shors TJ, Townsend DA, Zhao M, Kozorovitskiy Y, Gould E (2002). Neurogenesis may relate to some but not all types of hippocampal-dependent learning.. Hippocampus.

[5] Snyder JS, Hong NS, McDonald RJ, Wojtowicz JM (2005). A role for adult neurogenesis in spatial long-term memory.. Neuroscience.

[6] Zhang CL, Zou Y, He W, Gage FH, Evans RM (2008). A role for adult TLX-positive neural stem cells in learning and behavior.. Nature.

[7] Dupret D, Revest JM, Koehl M, Ichas F, De Giorgi F (2008). Spatial relational memory requires hippocampal adult neurogenesis.. PLoS ONE.

[8] Dupret D, Fabre A, Dobrossy MD, Panatier A, Rodriguez JJ (2007). Spatial learning depends on both the addition and removal of new hippocampal neurons.. PLoS Biol.

[9] Kempermann G, Gage FH (2002). Genetic determinants of adult hippocampal neurogenesis correlate with acquisition, but not probe trial performance in the water maze task.. Eur J Neurosci.

[10] Kempermann G (2002). Why new neurons? Possible functions for adult hippocampal neurogenesis.. J Neurosci.

[11] Kempermann G (2008). The neurogenic reserve hypothesis: what is adult hippocampal neurogenesis good for?. Trends Neurosci.

[12] Wiskott L, Rasch MJ, Kempermann G (2006). A functional hypothesis for adult hippocampal neurogenesis: avoidance of catastrophic interference in the dentate gyrus.. Hippocampus.

[13] Wolfer DP, Mohajeri HM, Lipp HP, Schachner M (1998). Increased flexibility and selectivity in spatial learning of transgenic mice ectopically expressing the neural cell adhesion molecule L1 in astrocytes.. Eur J Neurosci.

[14] Brody DL, Holtzman DM (2006). Morris water maze search strategy analysis in PDAPP mice before and after experimental traumatic brain injury.. Exp Neurol.

[15] Wolfer DP, Madani R, Valenti P, Lipp HP (2001). Extended analysis of path data from mutant mice using the public domain software Wintrack.. Physiol Behav.

[16] Wolfer DP, Lipp HP (2000). Dissecting the behaviour of transgenic mice: is it the mutation, the genetic background, or the environment?. Exp Physiol.

[17] Balschun D, Wolfer DP, Gass P, Mantamadiotis T, Welzl H (2003). Does cAMP response element-binding protein have a pivotal role in hippocampal synaptic plasticity and hippocampus-dependent memory?. J Neurosci.

[18] Chowdhury S, Vaughan MM, Gore ME (1999). New approaches to the systemic treatment of melanoma.. Cancer Treat Rev.

[19] EMEA (2005).

[20] Mason WP (2005). Progress in clinical neurosciences: Advances in the management of low-grade gliomas.. Can J Neurol Sci.

[21] Kee N, Teixeira CM, Wang AH, Frankland PW (2007). Preferential incorporation of adult-generated granule cells into spatial memory networks in the dentate gyrus.. Nat Neurosci.

[22] Wang S, Scott BW, Wojtowicz JM (2000). Heterogenous properties of dentate granule neurons in the adult rat.. J Neurobiol.

[23] Schmidt-Hieber C, Jonas P, Bischofberger J (2004). Enhanced synaptic plasticity in newly generated granule cells of the adult hippocampus.. Nature.

[24] Wang JW, David DJ, Monckton JE, Battaglia F, Hen R (2008). Chronic fluoxetine stimulates maturation and synaptic plasticity of adult-born hippocampal granule cells.. J Neurosci.

[25] Kempermann G, Gage FH (2002). Genetic influence on phenotypic differentiation in adult hippocampal neurogenesis.. Brain Res Dev Brain Res.

[26] Goodrich-Hunsaker NJ, Hunsaker MR, Kesner RP (2008). The interactions and dissociations of the dorsal hippocampus subregions: how the dentate gyrus, CA3, and CA1 process spatial information.. Behav Neurosci.

[27] Steele RJ, Morris RG (1999). Delay-dependent impairment of a matching-to-place task with chronic and intrahippocampal infusion of the NMDA-antagonist D-AP5.. Hippocampus.

[28] Saxe MD, Malleret G, Vronskaya S, Mendez I, Garcia AD (2007). Paradoxical influence of hippocampal neurogenesis on working memory.. Proc Natl Acad Sci U S A.

[29] Brandeis R, Brandys Y, Yehuda S (1989). The use of the Morris Water Maze in the study of memory and learning.. Int J Neurosci.

[30] Aimone JB, Wiles J, Gage FH (2006). Potential role for adult neurogenesis in the encoding of time in new memories.. Nat Neurosci.

[31] Aimone JB, Wiles J, Gage FH (2009). Computational influence of adult neurogenesis on memory encoding.. Neuron.

[32] Becker S (2005). A computational principle for hippocampal learning and neurogenesis.. Hippocampus.

[33] Becker S, Wojtowicz JM (2007). A model of hippocampal neurogenesis in memory and mood disorders.. Trends Cogn Sci.

[34] Chambers RA, Potenza MN, Hoffman RE, Miranker W (2004). Simulated apoptosis/neurogenesis regulates learning and memory capabilities of adaptive neural networks.. Neuropsychopharmacology.

[35] Deisseroth K, Singla S, Toda H, Monje M, Palmer TD (2004). Excitation-neurogenesis coupling in adult neural stem/progenitor cells.. Neuron.

[36] Feng R, Rampon C, Tang YP, Shrom D, Jin J (2001). Deficient Neurogenesis in Forebrain-Specific Presenilin-1 Knockout Mice Is Associated with Reduced Clearance of Hippocampal Memory Traces.. Neuron.

[37] Kronenberg G, Reuter K, Steiner B, Brandt MD, Jessberger S (2003). Subpopulations of proliferating cells of the adult hippocampus respond differently to physiologic neurogenic stimuli.. J Comp Neurol.

[38] Seri B, Garcia-Verdugo JM, McEwen BS, Alvarez-Buylla A (2001). Astrocytes give rise to new neurons in the adult mammalian hippocampus.. J Neurosci.

[39] Le Marec N, Caston J, Lalonde R (1997). Impaired motor skills on static and mobile beams in lurcher mutant mice.. Exp Brain Res.

[40] Walsh RN, Cummins RA (1976). The Open-Field Test: a critical review.. Psychol Bull.

[41] Morris R (1984). Developments of a water-maze procedure for studying spatial learning in the rat.. J Neurosci Methods.

[42] Janus C (2004). Search strategies used by APP transgenic mice during navigation in the Morris water maze.. Learn Mem.

